# Supplementary figures and images for: Diagnosis of fasciolosis antibodies in Brazilian cattle through ELISA employing both native and recombinant antigens
Source: Microbiol Spectr. 2024 Mar 27;12(5):e00095-24. doi: 10.1128/spectrum.00095-24 (PMC11064638; doi:10.1128/spectrum.00095-24)

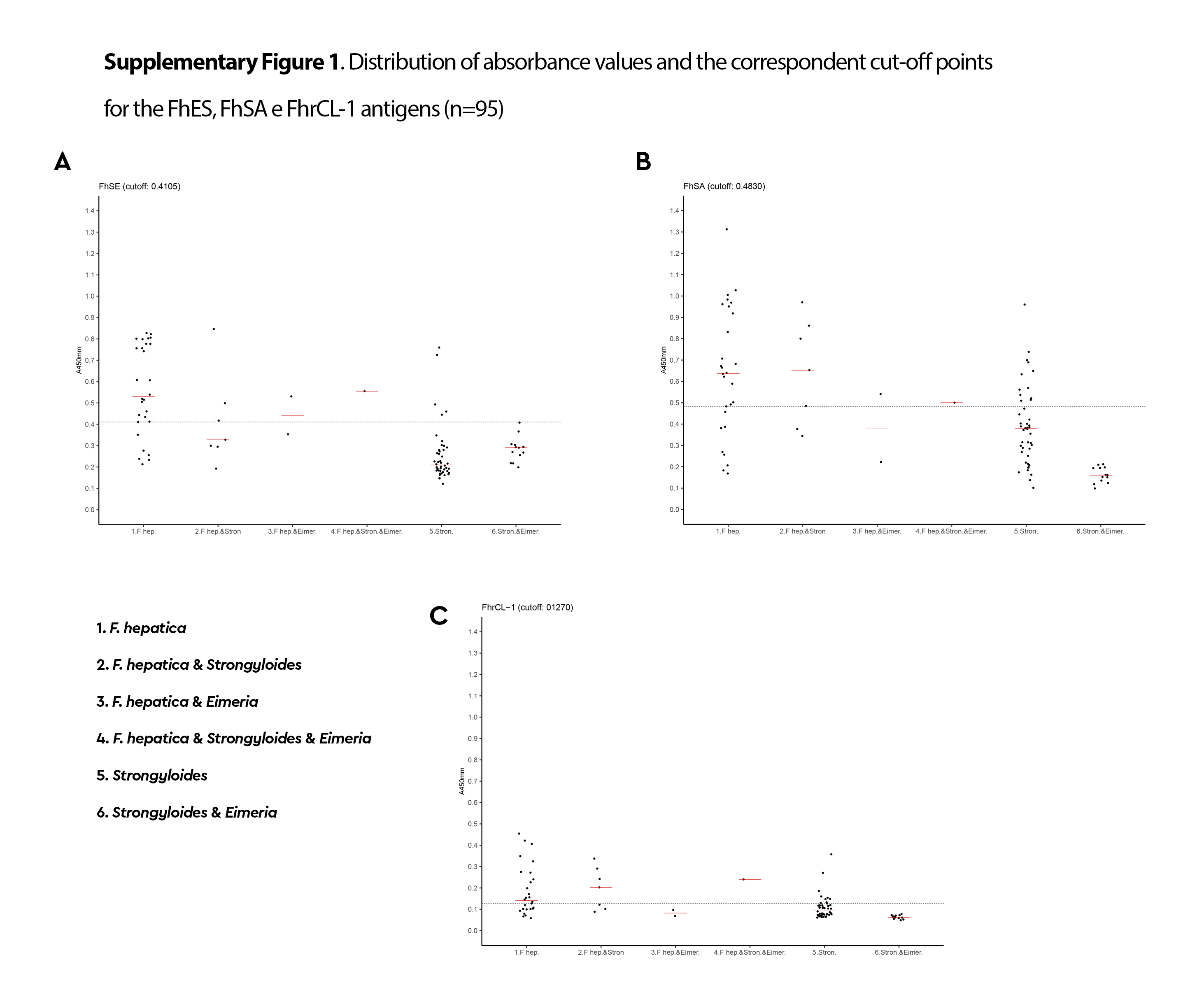

Supplement: Figure S1 — Distribution of absorbance values. [file spectrum.00095-24-s0001.tiff]

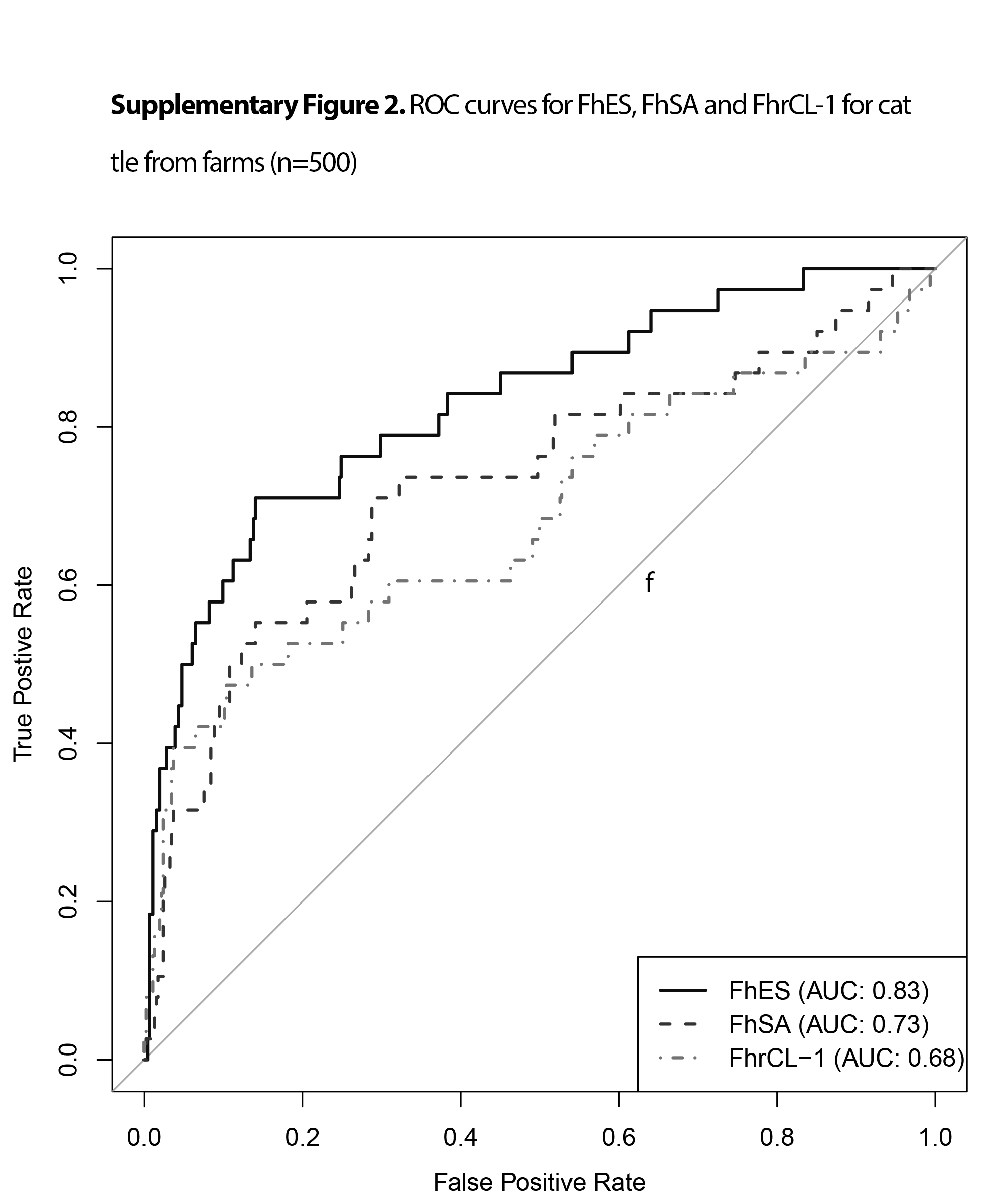

Supplement: Figure S2 — ROC curves. [file spectrum.00095-24-s0002.tiff]
