# Supplementary material for: Diagnosis of fasciolosis antibodies in Brazilian cattle through ELISA employing both native and recombinant antigens
Source: Microbiol Spectr. 2024 Mar 27;12(5):e00095-24. doi: 10.1128/spectrum.00095-24 (PMC11064638; doi:10.1128/spectrum.00095-24)
Supplement: Tables S1 and S2 — Diagnostic performance measures for the three tests on farms cattle (n = 500, 38 positive cases). [file spectrum.00095-24-s0003.docx]

**Supplementary Table 1.** Descriptive summary for the three tests when applied to cattle from farms (n=500).

|  | **Native antigens (OD)** | | **Recombinant antigen (OD)** |
| --- | --- | --- | --- |
| **Summary**  **Values** | ***Fh*ES** | ***Fh*SA** | ***Fh*rCL-1** |
| **Positive group (n= 38)** |  |  |  |
| Minimum | 0.192 | 0.168 | 0.058 |
| 1st quartile | 0.352 | 0.405 | 0.101 |
| Median | 0.510 | 0.629 | 0.141 |
| Mean (SD) | 0.526 (0.211) | 0.624 (0.284) | 0.183 (0.110) |
| 2nd quartile | 0.756 | 0.854 | 0.242 |
| Maximum | 0.847 | 1.312 | 0.454 |
| **Negative group (n= 462)** |  |  |  |
| Minimum | 0.085 | 0.078 | 0.048 |
| 1st quartile | 0.199 | 0.253 | 0.085 |
| Median | 0.256 | 0.378 | 0.101 |
| Mean (SD) | 0.285 (0.122) | 0.397 (0.192) | 0.111 (0.046) |
| 2nd quartile | 0.346 | 0.507 | 0.125 |
| Maximum | 0.898 | 1.373 | 0.410 |

Legend: SD= Standard Deviation; OD= Optical Density.

**Supplementary Table 2.** Diagnostic performance measures for the three tests on farms cattle considering coprological examination as the gold standard method (n=500, 38 positive cases).

|  | **Native antigens** | | **Recombinant antigen** |
| --- | --- | --- | --- |
| **Performance measures** | ***Fh*ES**  **estimate**  **(95%CI)** | ***Fh*SA**  **estimate**  **(95%CI)** | ***Fh*rCL-1**  **estimate**  **(95%CI)** |
| AUC ROC curve | 0.83 (0.75 - 0.90) | 0.73 (0.63 - 0.83) | 0.68 (0.58 - 0.79) |
| Cutoff | 0.4060 (OD) | 0.4750 (OD) | 0.1175 (OD) |
| Sensitivity | 27/38 (0.71)  (0.55 - 0.83) | 27/38 (0.71)  (0.55 - 0.83) | 23/38 (0.61)  (0.44 - 0.75) |
| Specificity | 396/462 (0.86)  (0.82 - 0.89) | 328/462 (0.71)  (0.67 - 0.75) | 318/462 (0.69)  (0.64 - 0.73) |
| PPV | 27/93 (0.29)  (0.23 - 0.36) | 27/161 (0.17)  (0.14 - 0.21) | 23/167 (0.14)  (0.11 - 0.18) |
| NPV | 396/407 (0.97)  (0.96 - 0.98) | 328/339 (0.97)  (0.95 - 0.98) | 318/333 (0.95)  (0.93 - 0.97) |

Legend: CI= Confidence Intervals; PPV= Positive Predictive Values; NPV= Negative Predictive Values; OD= Optical Density.
